# Platelet-Rich Plasma (PRP) Assessment in Degenerative Discovertebral Complexes (DVC) Using Quantitative MRI at 4.7T: A Preliminary Animal In Vivo Study

**DOI:** 10.3390/bioengineering13080909

**Published:** 2026-08-11

**Authors:** Benjamin Dallaudière, Emeline J. Ribot, Laurence Dallet, Aurélien J. Trotier, Olivier Thibaudeau, Sylvain Miraux, Olivier Hauger

**Affiliations:** 1 Department of Radiology, University Hospital of Bordeaux, 33076 Bordeaux, France; olivier.hauger@chu-bordeaux.fr; 2 Centre de Résonance Magnétique des Systèmes Biologiques (CRMSB), CNRS, University Bordeaux, UMR5536, 33076 Bordeaux, France; ribot@rmsb.u-bordeaux.fr (E.J.R.); trotier@rmsb.u-bordeaux.fr (A.J.T.); sylvain.miraux@rmsb.u-bordeaux.fr (S.M.); 3 Plateforme d’Imagerie Biomédicale pIBIO, CNRS, University Bordeaux, UAR3767, 33076 Bordeaux, France; laurence.dallet@rmsb.u-bordeaux.fr; 4 Plateforme HIPNO, Inserm, US 34 Claude Bernard, Site Bichat, Université Paris Cité, 75018 Paris, France; olivier.thibaudeau@inserm.fr

**Keywords:** PRP, degenerative disc, MRI, small-animal

## Abstract

This exploratory in vivo study investigated the therapeutic potential of intradiscal platelet-rich plasma (PRP) in a rat model of degenerative disc disease (DDD) using quantitative 4.7-T MRI and histologic correlation. Eight female Sprague-Dawley rats underwent induction of disc–vertebral complex degeneration through combined mechanical injury and type I collagenase injection. Immediately thereafter, a single intradiscal injection of leukocyte-poor PRP was administered. Longitudinal MRI evaluation included 3D ultrashort echo time (UTE) imaging, T1 mapping, and T2 mapping at baseline and during follow-up. In untreated discs, degeneration was associated with progressive decreases in nucleus pulposus T1 and T2 values and increased annulus fibrosus T2 values, reflecting dehydration and structural disorganization. In contrast, PRP-treated discs showed relative preservation of nucleus pulposus T1 and T2 relaxation times, while annulus fibrosus T2 values remained more stable, suggesting attenuation of degenerative changes. UTE-derived signal changes were less discriminatory between treated and untreated groups. Histologic analysis confirmed severe nucleus pulposus and annulus fibrosus disorganization in untreated discs, whereas PRP-treated discs demonstrated milder alterations with preserved vertebral endplate architecture. Overall, these preliminary findings suggest that the longitudinal effect of a treatment through intradiscal PRP is feasible and this injection may modulate early degenerative changes in the current animal model. However, the results should be interpreted with caution because of the exploratory design, the aggressive animal degeneration model, the absence of a sham-injection comparator group, and the small number of animals studied.

## 1. Introduction

The intervertebral disc is a key component of the disc–vertebral complex (DVC), which also includes the vertebral endplates and adjacent vertebral bodies. Pathologies affecting the DVC encompass intervertebral disc degeneration, disc herniation, Modic changes, endplate defects, and chronic low back pain, all of which represent major contributors to disability worldwide. The etiology of these disorders is multifactorial and involves aging, genetic predisposition, mechanical overloading, trauma, smoking, obesity, metabolic disorders, and inflammatory processes, which collectively contribute to extracellular matrix degradation, altered disc biomechanics, and progressive structural failure. Epidemiologically, intervertebral disc degeneration is highly prevalent, affecting the majority of adults to varying degrees with increasing age, while low back pain remains the leading cause of years lived with disability globally. The substantial socioeconomic burden associated with DVC pathologies highlights the need for improved diagnostic tools and effective regenerative therapeutic strategies.

Platelet-rich plasma (PRP) is an autologous blood-derived concentrate enriched in platelets and endogenous growth factors. It has been widely investigated in orthopedics and sports medicine for conditions involving tendon, ligament, cartilage, muscle, and bone healing, and it is increasingly explored in spine and neurosurgical applications, including chronic low back pain and intervertebral disc degeneration [1,2,3,4,5,6,7,8]. Its biological rationale relies on the local release of platelet-derived mediators that may influence inflammation, extracellular matrix turnover, angiogenesis, and tissue repair.

In current musculoskeletal practice, PRP is mainly used or investigated for tendinopathies, ligament injuries, osteoarthritis, cartilage lesions, muscle injuries, and selected bone-healing disorders. In the spine, intradiscal PRP remains investigational, but it has attracted interest as a minimally invasive biological strategy aimed at reducing symptoms and potentially slowing degenerative changes in selected patients with discogenic low back pain. Available clinical data remain limited, heterogeneous, and mostly focused on pain and function rather than imaging-based structural endpoints [3,4,5,6,7,8].

Experimental in vitro studies investigating the effects of PRP on disc–vertebral complex (DVC) cells have yielded encouraging but heterogeneous results, suggesting a potential therapeutic role for PRP in DVC degeneration. Akeda et al. demonstrated that PRP-derived soluble releasate stimulated cell proliferation and increased proteoglycan and collagen synthesis in alginate-cultured porcine DVC cells without altering phenotypic stability, with greater stimulatory effects observed in the annulus fibrosus (AF) compared with the nucleus pulposus (NP) [2,9]. Similarly, Chen et al. reported PRP-induced NP cell proliferation, enhanced three-dimensional tissue formation, upregulation of matrix-associated genes, and increased glycosaminoglycan accumulation [10]. These observations support the concept that PRP may influence both cellular activity and extracellular matrix metabolism within the disc.

Platelets contribute to tissue repair not only through hemostasis but also via the release of growth factors following activation, promoting inflammation, angiogenesis, and tissue regeneration during wound healing. Hondke et al. showed that PRP enhanced migration and viability of AF cells in both early and advanced stages of degeneration, with a stronger response observed in early-stage AF cells [11].

Conversely, Mietsch et al. reported that although PRP stimulated cell proliferation in NP and mesenchymal stem cell (MSC) cultures, it did not induce chondrogenic differentiation compared with transforming growth factor beta-1 (TGF-β1), leading the authors to question the suitability of PRP for human NP tissue engineering applications [12].

Findings from in vivo studies largely corroborate in vitro data, demonstrating restorative effects of PRP in joint and musculoskeletal degeneration models. However, variability in outcomes is common due to limited standardization of PRP preparation and activation protocols. In addition, several animal studies have investigated PRP in combination with other agents to elucidate the mechanisms underlying its potential to delay intervertebral disc (IVD) degeneration [2]. Gullung et al., for example, reported preservation of disc morphology, hydration, and disc height following PRP injection in a rat lumbar disc needle puncture model [13].

Recent advances in quantitative MRI have enabled improved assessment of disc degeneration and therapeutic response beyond conventional morphologic grading. Conventional T2-weighted imaging and disc-height measurements remain useful but are relatively insensitive to early biochemical changes. Quantitative T2 mapping is sensitive to water content and collagen/proteoglycan matrix organization, whereas T1 mapping may reflect tissue composition, hydration, and matrix remodeling. Ultrashort echo time (UTE) imaging provides access to tissues with very short T2 components and may improve visualization of collagen-rich structures such as the annulus fibrosus, endplate region, and fibrocartilaginous components. Dallaudière et al. demonstrated that 3D UTE imaging combined with T1 and T2 mapping allowed comprehensive visualization of DVC anatomy in a rat model. Quantitative analysis of signal-to-noise and contrast-to-noise ratios in the AF and NP reliably differentiated healthy discs from surgically induced DDD. T2 mapping revealed increased T2 values in the AF and decreased values in the NP at postoperative weeks 1 and 2, while T1 mapping demonstrated a significant reduction in T1 values in both compartments over the same period. These quantitative changes were consistent with findings obtained using 3D-UTE imaging [14,15].

Given the growing interest in PRP-based therapies and the increasing availability of quantitative MRI biomarkers, further studies integrating standardized PRP protocols with advanced imaging techniques are warranted. The objective of this exploratory study was to assess whether intradiscal PRP injection could attenuate experimentally induced DDD in vivo using longitudinal quantitative 4.7-T MRI and histologic correlation. We hypothesized that PRP-treated discs would show relative preservation of NP T1 and T2 relaxation times, more stable AF T2 values, and milder histologic disorganization compared with untreated degenerated discs. We further hypothesized that T1 and T2 mapping would be more sensitive than UTE-derived signal metrics for detecting treatment-related changes.

## 2. Material and Methods

### 2.1. Animal Model

All procedures and animal care complied with the European Union Directive on the protection of animals used for scientific purposes and were approved by the French Ministry of Agriculture (#20061). This exploratory longitudinal study included eight female immunocompetent Sprague-Dawley rats (Janvier Labs, Saint Berthevin, France), aged 8 weeks (mean body weight, 184 +/− 21 g), corresponding to 16 analyzed DVCs at baseline. The sample size (n = 8) was intentionally limited in accordance with the 3R principles for animal research, as the surgical procedure was classified as severe, and because each animal underwent repeated longitudinal MRI examinations, thereby increasing the number of repeated measurements while reducing the number of animals required. Animals were housed in standard cages under controlled environmental conditions (12-h light/dark cycle; temperature, 20 +/− 2 degrees C).

Degenerative disc disease (DDD) of the disc–vertebral complex (DVC) was induced using a combined mechanical and chemical approach. Intradiscal trituration was performed with an 18-gauge needle, followed by a single intradiscal injection of type I collagenase (Gibco, Illkirch, France; 20% solution in 100 µL saline), administered by the same operator (BD) [15]. This mechanical/chemical model has been shown to be simpler yet equally effective compared with surgical or infectious models and results in reproducible DDD as early as 3 days after treatment [16,17,18,19].

Animals were clinically monitored every 2 days for changes in body weight and general behavior.

No formal a priori power calculation was performed because this work was designed as a preliminary exploratory animal study intended to assess technical feasibility, longitudinal quantitative MRI behavior, and histologic correlation after intradiscal PRP injection. The sample size was deliberately limited in accordance with the 3R principles for animal research. The repeated-measures design, with longitudinal imaging of the same animals over time and analysis of treated and adjacent nondegenerated DVCs, was used to reduce interindividual variability; nevertheless, we acknowledge that the limited cohort size reduces statistical power and restricts the generalizability of the findings.

### 2.2. PRP Preparation and Intradiscal Injection

PRP treatment consisted of a single intradiscal injection administered immediately after DDD induction. PRP preparation, injection, and evaluation were performed by the same operators (BD and ER).

Under anesthesia, 1 mL of whole blood was collected from the caudal vein using a syringe and transferred into anticoagulant tubes containing 0.1 mL of 10% sodium citrate. Samples were centrifuged at 220× *g* for 20 min at room temperature. After centrifugation, two layers were identified: a lower erythrocyte fraction and an upper plasma fraction. The buffy coat, located at the interface between these layers and enriched in platelets and leukocytes, was included in the supernatant. This supernatant was collected and centrifuged again at 480× *g* for 20 min at 4 °C. The resulting pellet, corresponding to platelet-rich plasma (PRP), was extracted.

**Figure 1:**
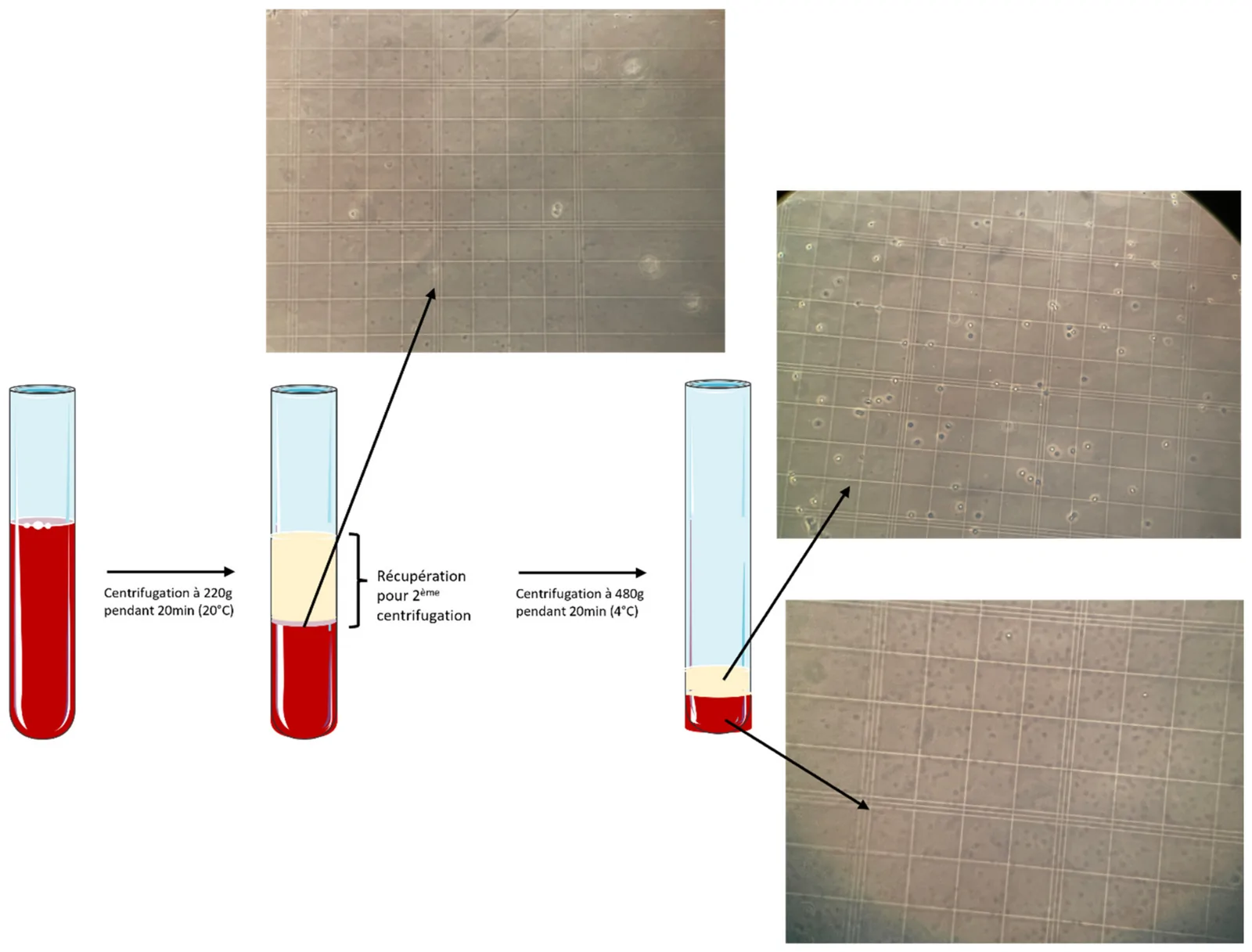
PRP creation using a Neubauer counting chamber. Platelets were manually counted in four grid lines.

Platelet concentration was determined using a Thrombo-TIC kit (Labelians Bioanalytic GmbH, Nemours, France). Briefly, 10 µL of PRP was diluted in 900 µL of Thrombo-TIC reagent and incubated at room temperature for 5 min. A 15-µL aliquot was then loaded onto a Neubauer counting chamber. After sedimentation for 15 min in a humidified chamber, platelets were manually counted in four grid lines. Platelet concentration (platelets/L) was calculated using the following formula:

(Number of platelets × 100 × 10^6^)/(80 × 0.00025 × 0.02) (Figure 1).

Nonactivated PRP was injected intradiscally within 30 min of preparation using a 21-gauge needle. Correct intradiscal positioning was confirmed in real time by the absence of resistance during injection. Animals were monitored for 2 h post-procedure to detect any adverse events.

PRP characterization was restricted to platelet concentration and leukocyte-poor classification. Growth factor concentrations, cytokine profiles, platelet activation markers, and intersample biologic variability were not measured in the present study. To improve reproducibility despite this limitation, all preparations followed the same centrifugation protocol, anticoagulant conditions, counting method, injection timing, and operator workflow.

### 2.3. MRI Acquisition

In vivo MRI of the DVC was performed at baseline (day 0 [D0], n = 8 animals; 16 DVCs) and weekly for 6 weeks following DDD induction using a 4.7-T Bruker BioSpec system (Ettlingen, Germany). The system was equipped with gradients providing a maximum strength of 660 mT/m and a rise time of 110 µs. A volume resonator (inner diameter, 75.4 mm; active length, 70 mm) operating in quadrature mode was used for radiofrequency transmission, and a four-element (2 × 2) phased-array surface coil was used for signal reception.

Following induction of anesthesia, animals were positioned prone in the MRI scanner with the proximal tail centered within the radiofrequency coil. Respiratory activity was continuously monitored during image acquisition.

The MRI protocol included a three-dimensional ultrashort echo time (3D-UTE) sequence, a parametric two-dimensional T2 mapping sequence, and a three-dimensional T1 mapping sequence with the following parameters:

The selected imaging parameters were chosen to balance spatial resolution, quantitative robustness, and acceptable acquisition time for repeated in vivo examinations. The high-field 4.7-T system and small field of view allowed submillimetric evaluation of the rat DVC. UTE imaging was included to assess short-T2 components of the disc and endplate region, whereas T1 and T2 mapping were selected as quantitative biomarkers of disc composition, hydration, and matrix organization.

3D-UTE sequence: TR/TE, 11.863/0.031 ms; field of view (FOV), 17 × 17 × 17 mm; matrix, 128 × 128 × 128; isotropic spatial resolution, 0.133 mm; number of excitations, 3; number of projections, 51,360; bandwidth, 100 kHz; flip angle, 10°; hard excitation pulse duration, 0.05 ms; fat saturation module (bandwidth, 701.19 Hz; duration, 7.6 ms; flip angle, 90°); acquisition time, 30 min 28 s.

2D T2 mapping (MSME sequence): Sagittal orientation; 100 echoes acquired from minimum TE (3.8 ms) to 378.6 ms; TR, 8000 ms; FOV, 20 × 15 mm; matrix, 128 × 96; in-plane resolution, 0.156 mm; 20 slices; slice thickness, 0.6 mm; number of excitations, 1; bandwidth, 71.4 kHz; total acquisition time, 12 min 48 s.

3D T1 mapping (MP2RAGE sequence): TI1/TI2/MP2RAGE TR, 800/2200/6250 ms; FOV, 22.5 × 22.5 × 11.25 mm; matrix, 128 × 128 × 64; isotropic spatial resolution, 0.176 mm; inversion pulse, hyperbolic secant (10 ms); flip angles, 7°/7°; TR/TE, 6/2.16 ms; receiver bandwidth, 50 kHz; number of excitations, 4; acquisition time, 21 min 15 s [20,21].

**Figure 2:**
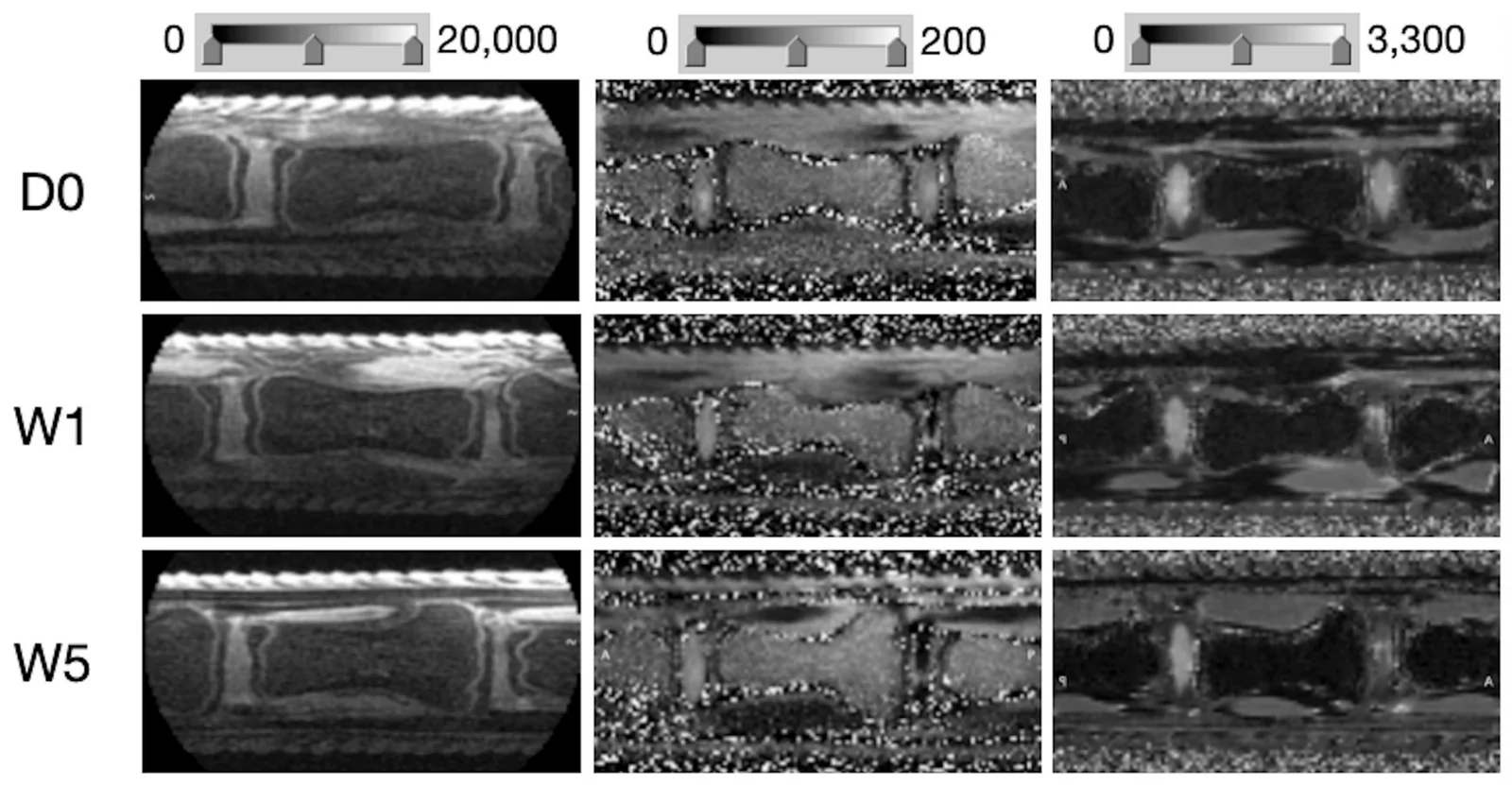
Sagittal 3D UTE-weighted, T1 and T2 acquisition images of the discovertebral complex (DVC) of untreated rats during in vivo assessment at day 0 (D0) and week (W)1 and W5. The units of the parametric maps are in ms.

**Figure 3:**
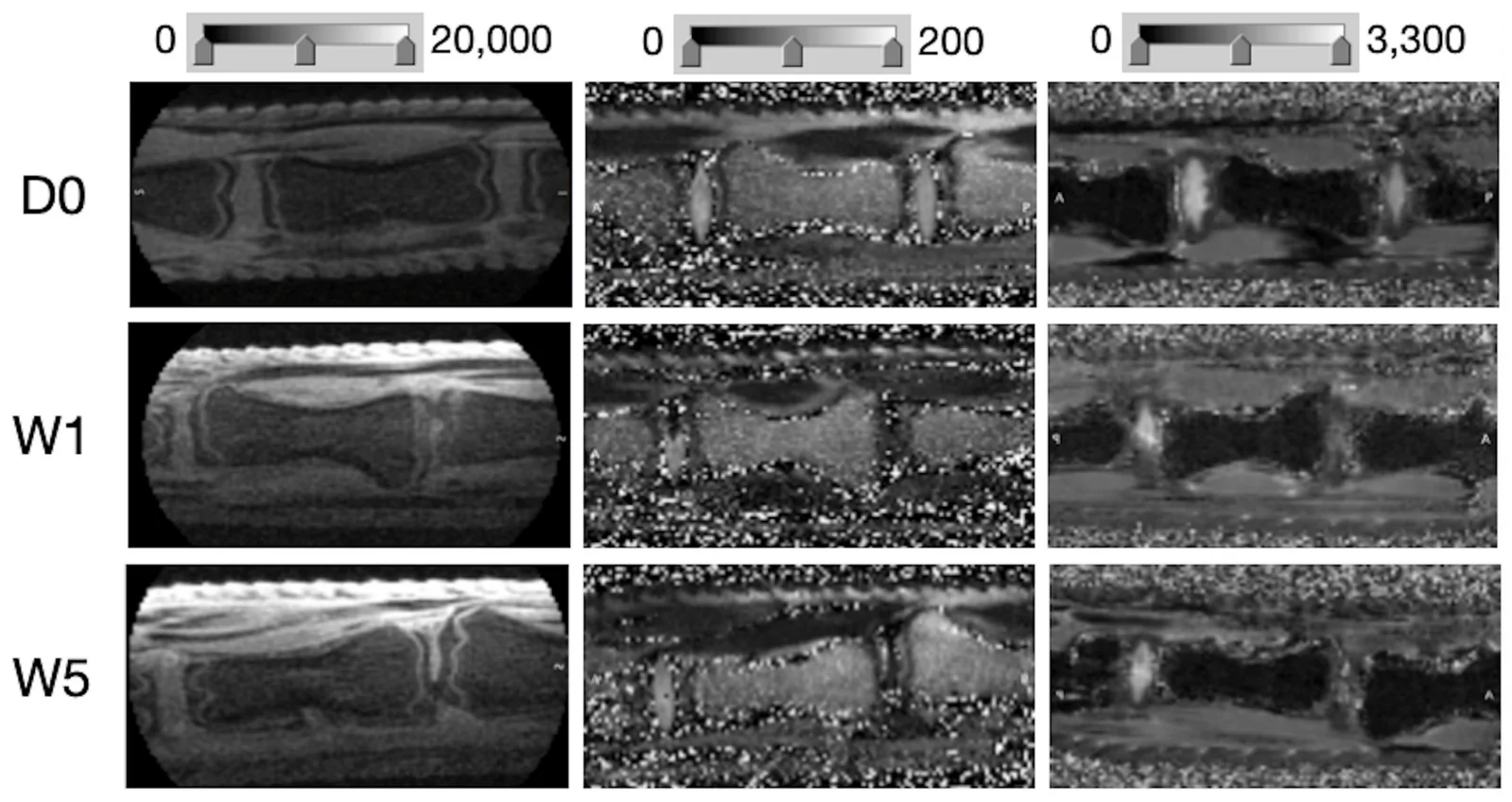
Sagittal 3D UTE-weighted, T1 and T2 acquisition sequences of the discovertebral complex (DVC) of treated rats during in vivo assessment at day 0 (D0) and week (W)1 and W5. The units of the parametric maps are in ms.

The mean total examination time, including animal positioning, was approximately 65 min. At the end of the longitudinal follow-up, tail specimens were harvested, fixed in 4% paraformaldehyde overnight, and stored in phosphate-buffered saline (Figure 2 and Figure 3).

### 2.4. Image Analysis

Image and quantitative map reconstruction was performed using MATLAB-based software (R2024a, MathWorks). Qualitative assessment, quantitative signal intensity analysis from 3D-UTE images, and T1 and T2 quantification were independently performed by a musculoskeletal radiologist and a physicist (BD and ER), each with over 10 years of experience. Measurements were performed using a standardized ROI-placement strategy on the central sagittal section of each DVC. Interobserver discrepancies were resolved by consensus.

For quantitative analysis, two regions of interest (ROIs; six pixels each) were placed on sagittal images within the nucleus pulposus (NP), annulus fibrosus (AF), cartilaginous endplate (CEP), growth plate (GP), and subchondral bone (SB) in both normal and degenerative DVCs. Considering the acquisition spatial resolution of the different sequences applied, the approximate physical volumes of each ROI were 0.014 mm^3^, 0.088 mm^3^ and 0.033 mm^3^ for the UTE, T2 and T1 maps, respectively. Degenerated DVCs and adjacent nondegenerated control DVCs were analyzed.

Results were expressed as signal-to-noise ratio (SNR) for 3D-UTE imaging, and as mean T2 and T1 relaxation times ± standard deviation. SNR was calculated as the mean signal intensity within an ROI divided by the mean background noise magnitude corrected by a factor of 2.74 to account for the Rayleigh noise distribution associated with the four-element phased-array coil [22].

### 2.5. Histopathologic Analysis

Following tail harvesting, spinal segments were decalcified, sectioned in the midsagittal plane, dehydrated in 75% ethanol, and embedded in paraffin. Sections (5 µm thick) were stained with hematoxylin and eosin and Masson trichrome. Histologic analysis was performed in the sagittal plane to ensure optimal correlation with MRI findings. All DVC components (NP, AF, CEP, GP, and SB) were evaluated, focusing on NP disorganization, AF fissuring, endplate disruption, and SB edema and neovascularization.

### 2.6. Statistical Analysis

Statistical analyses were performed using GraphPad Prism software (version 10). Data normality was assessed using the Shapiro–Wilk test. Homogeneity of variance was assessed before parametric comparisons [to be completed according to Prism output: Brown–Forsythe or Bartlett test]. Depending on data distribution, either a parametric *t* test or a nonparametric Mann–Whitney test was used to compare quantitative MRI parameters across DVC compartments between PRP-treated and untreated animals.

For longitudinal comparisons of relaxation times within one structure (AF or NP), one-way ANOVA with repeated measures followed by Tukey post hoc multiple-comparison testing was used when assumptions of normality and homoscedasticity were satisfied. When these assumptions were not met, data were analyzed using the Kruskal–Wallis test followed by Dunn multiple-comparison testing. Exact *p* values, F statistics, and degrees of freedom were extracted from the statistical output. A *p* value < 0.05 was considered statistically significant.

## 3. Results

### 3.1. Biological Characteristics of Injected PRP

All animals survived the intradiscal procedure without complications. Peripheral blood collection was standardized to 1000 µL per animal, yielding a PRP volume of approximately 68 µL after platelet-poor plasma (PPP) separation. The mean platelet content per injection was 4.3 × 10^8^ platelets, corresponding to a concentration of 6.3 × 10^6^ platelets/µL.

No significant differences were observed in platelet doses administered across PRP injections, indicating reproducibility of the preparation protocol based on platelet concentration. This method produced a PRP with a high platelet concentration. According to the DEPA classification system, the resulting PRP was classified as autologous leukocyte-poor PRP (ABA, LP-PRP). Other PRP characteristics, including growth-factor content and activation-related biochemical markers, were not measured.

### 3.2. MRI Quantification and Evolution During PRP Treatment

**Figure 4:**
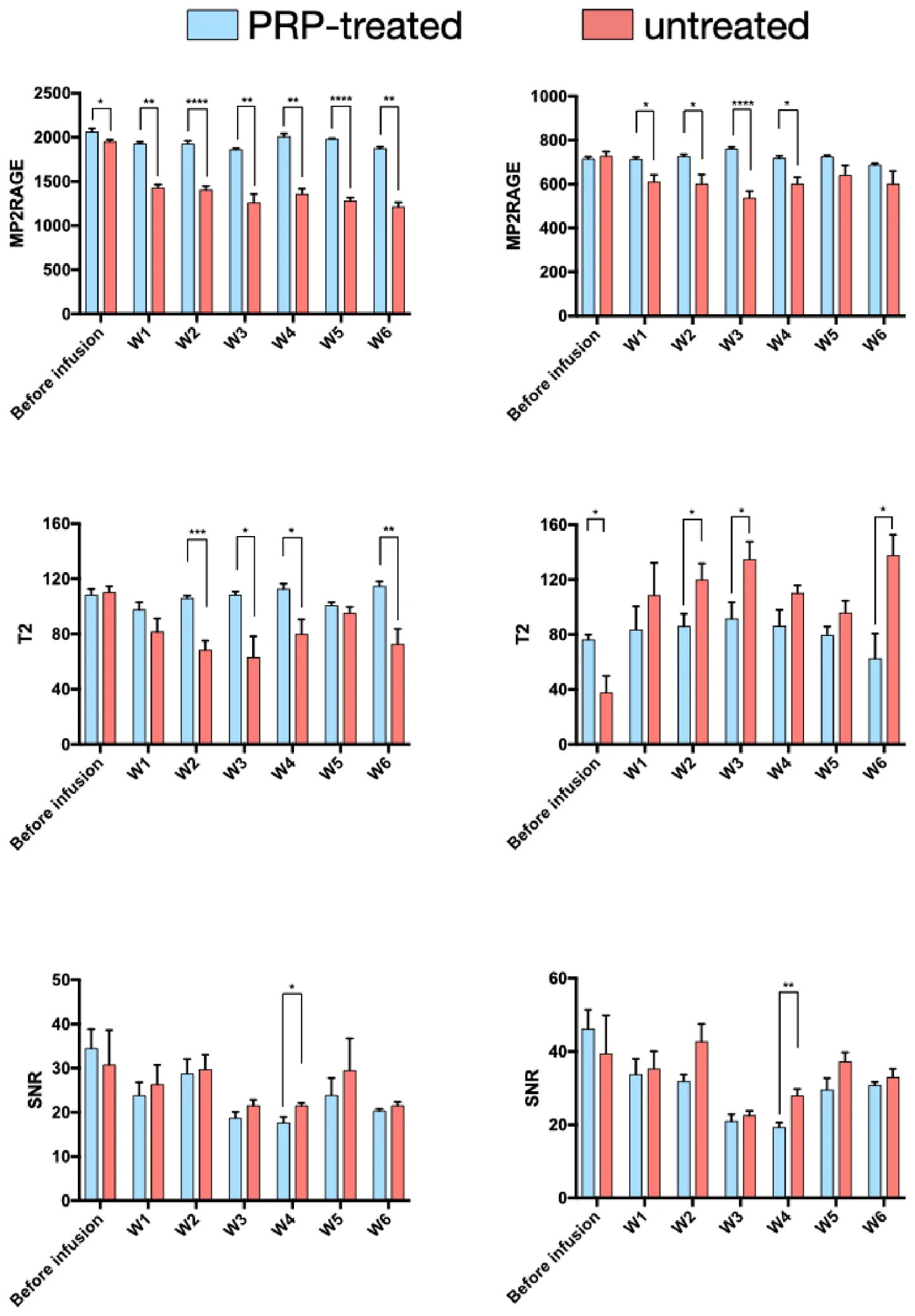
Main quantitative results of evolution of the annulus fibrosus (AF, left column) and nucleus pulposus (NP, right column) of the untreated and treated rat disc as determined from the 3D-UTE images, the T2 values (ms) and T1 values (ms) of the rat disc calculated from the T2 maps, and from the MP2RAGE maps. *, **, *** and **** represent *p* value < 0.05, 0.01, 0.001, 0.0001, respectively.

Quantitative imaging findings for healthy and degenerative discs are summarized in Figure 4.

**Table 1:** Summary table of the main results. df means “degree of freedom”.

Parameter	Main Finding and Statistics to Complete
UTE SNR	Limited discrimination between untreated and PRP-treated discs; significant differences only at W4 for selected AF (*p* = 0.0034, df = 8) and NP measurements (*p* = 0.0215, df = 8).
NP T1	Untreated discs showed persistent reduction (*p* = 0.002); PRP-treated discs returned toward baseline and differed from untreated discs at all time points (*p* < 0.04, df = 7–8).
AF T1	Untreated discs showed significant decrease only at W3; PRP-treated discs showed transient W3 increase with differences from untreated at W1–W4 (*p* < 0.02, df = 7–8).
NP T2	Untreated discs decreased, significantly at W3 (*p* = 0.03); PRP-treated discs showed relative preservation, differing from untreated at W2, W3, W4 and W6 (*p* < 0.02, df = 8).
AF T2	Untreated discs showed significant increase from W1–W6 (*p* = 0.007); PRP-treated discs remained relatively stable and were lower than untreated at W2, W3 and W6 (*p* < 0.04, df = 8).
Histology	Untreated discs showed severe NP/AF disorganization; PRP-treated discs showed milder disruption with preserved endplate architecture.

Quantitative analyses were restricted to the intervertebral disc compartments (annulus fibrosus [AF] and nucleus pulposus [NP]), as these structures constituted the primary targets of the degeneration model and were the only DVC components demonstrating temporal changes. No significant longitudinal variation was observed in the cartilaginous endplate (CEP), growth plate (GP), or subchondral bone (SB) in either semiquantitative or quantitative analyses.

#### 3.2.1. Quantitative MRI Analysis of Untreated DDD

In untreated DDD animals, ultrashort echo time (UTE) signal-to-noise ratio (SNR) values of both the AF and NP remained unchanged from week 1 (W1) through week 6 (W6) compared with baseline (D0) (all *p* > 0.05).

T1 relaxation times of the NP decreased significantly from baseline throughout follow-up (*p* = 0.002). AF T1 values tended to decrease after DDD induction but reached statistical significance only at W3 (*p* < 0.05).

AF T2 relaxation times increased significantly from baseline at all post-induction time points (W1–W6; *p* = 0.007). Similarly, NP T2 values were lower at all post-induction time points compared with D0, reaching statistical significance at W3 (*p* = 0.03).

#### 3.2.2. Quantitative MRI Analysis of PRP-Treated DDD and Comparison with Untreated DDD

In PRP-treated DDD animals, UTE SNR values of the AF decreased significantly from baseline but remained similar to those of untreated DDD animals at most time points, except at W4 (*p* = 0.0034, df = 8). NP UTE SNR values in PRP-treated animals were lower than baseline at all time points but reached statistical significance only at W4 (*p* = 0.036). Overall, UTE-derived signal changes showed limited ability to discriminate treated from untreated discs.

AF T1 relaxation times increased at W3 compared with baseline (F = (6,27)9.443, (*p* < 0.0001)) and subsequently returned toward baseline values. NP T1 values decreased until W3 (*p* = 0.0003) and then returned toward baseline. Importantly, NP T1 values differed significantly between treated and untreated animals at every time point, whereas AF T1 values differed between treated and untreated animals from W1 to W4 and became similar at W5 and W6.

NP T2 relaxation times did not significantly decrease from baseline in PRP-treated animals, except for selected inter-time comparisons between W1 and W4 and between W1 and W6 (F = (6,28)3.371, (*p* = 0.01)). NP T2 values differed significantly between treated and untreated animals at W2, W3, W4, and W6. AF T2 values remained relatively stable over time and tended to decrease after W3; AF T2 values were significantly lower in treated than untreated animals at W2, W3, and W6.

### 3.3. Histologic Evaluation

**Figure 5:**
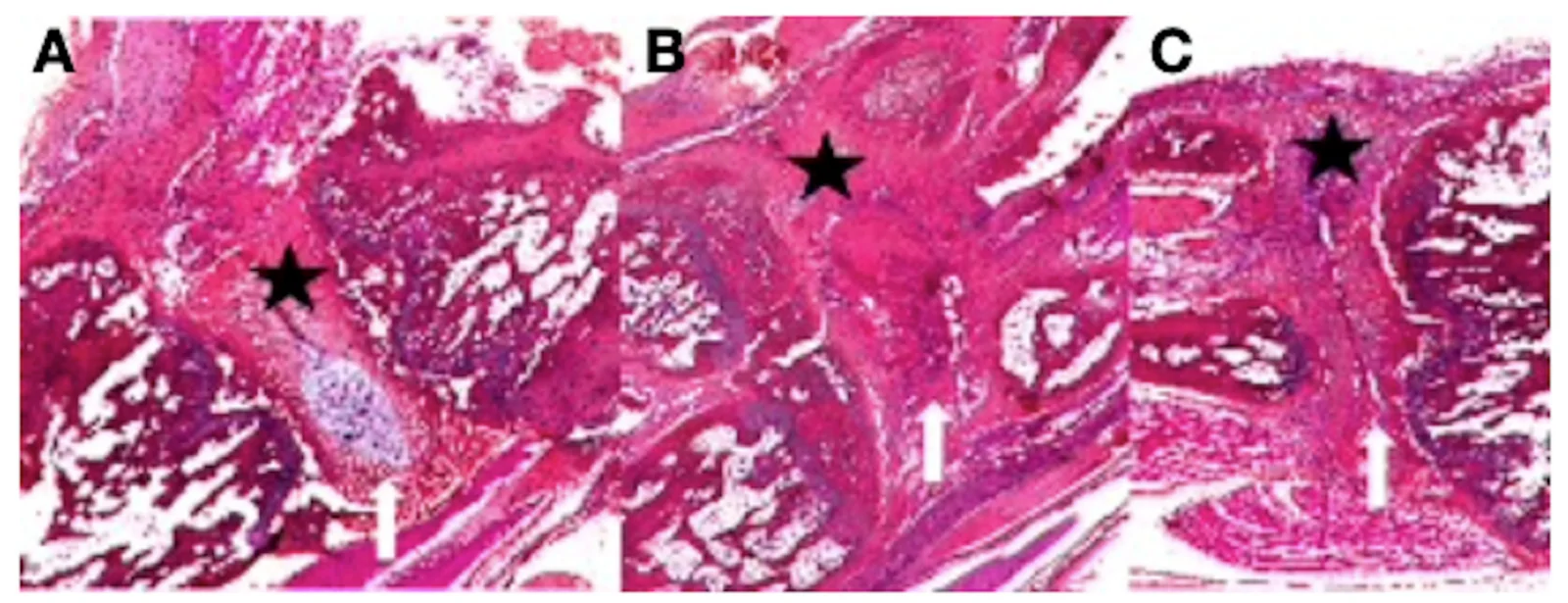
Sagittal DVC at D0 (**A**) and DDD histology at W2 (**B**) and W6 (**C**) with hematoxylin and eosin staining, showing progressive disorganization of the NP and AF. The vertebral endplate anatomy is unchanged. The arrows are pointing at the nucleus pulposus. The stars are located in the annulus.

**Figure 6:**
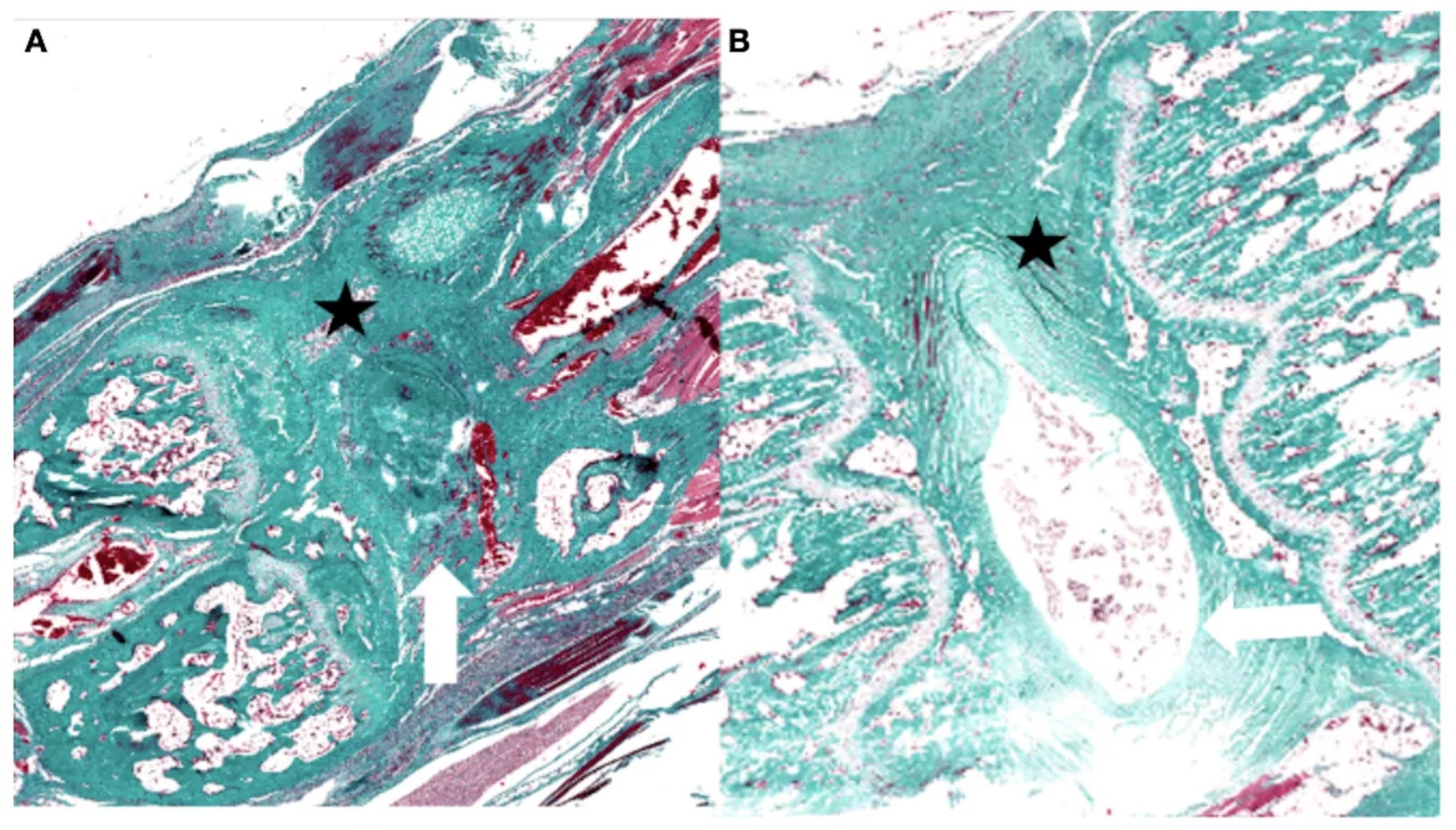
Sagittal DDD histology at W6 in untreated disc (**A**) and PRP-treated disc (**B**) with Masson trichrome staining, showing severe NP and AF disorganization in untreated DDD and milder NP and AF disruption in the treated disc. The vertebral endplate anatomy is unchanged. The arrows are pointing at the nucleus pulposus. The stars are located in the annulus.

Histologic examination of hematoxylin and eosin- and Masson trichrome-stained sections demonstrated the expected architecture of a healthy DVC, including the nucleus pulposus and annulus fibrosus, the cartilaginous and growth plate components of the endplate, and the subchondral bone. At week 2 after DDD induction, histologic findings revealed marked NP disruption, fibrillar disorganization of the AF, and a subtle reduction in disc height. In contrast, the endplate and subchondral bone appeared preserved, with no evidence of cartilaginous endplate alteration, growth plate fissuring, or subchondral bone edema (Figure 5 and Figure 6).

The MR quantitative and the qualitative histological results are summarized into Table 1.

## 4. Discussion

This exploratory study suggests that intradiscal injection of platelet-rich plasma (PRP) may attenuate early degenerative changes in a rat model of DDD. The effect was supported by quantitative MRI metrics derived from 3D T1 and 2D T2 mapping and by histologic analysis. In contrast, UTE-derived signal changes were less discriminatory between treated and untreated discs. The present results should therefore be interpreted as evidence of a potential protective or modulatory effect of PRP on early degeneration rather than as proof of true structural regeneration.

The current literature on regenerative medicine for lumbar degenerative disorders includes several systematic reviews and clinical studies evaluating biologic therapies for chronic low back pain. PRP has been investigated in orthopedics and sports medicine for tendon disorders, cartilage disease, osteoarthritis, muscle injuries, ligament healing, and selected bone-healing conditions. In neurosurgical and spine-related applications, intradiscal PRP remains at an investigational stage, with available studies primarily addressing pain, disability, and safety rather than quantitative imaging biomarkers or histologic endpoints [3,4,5,6,7,8].

With regard to in vivo rat models, only one randomized controlled animal study has previously evaluated intradiscal PRP injection [13]. In that study, lumbar intervertebral discs of Sprague-Dawley rats were injured by needle puncture and treated with either immediate PRP injection, delayed PRP injection, or sham intervention. Both PRP-treated groups demonstrated preservation of disc morphology, reduced inflammatory cell infiltration, and higher MRI-derived fluid content compared with sham-treated animals, with more pronounced effects observed following immediate PRP administration. Disc height was significantly preserved in the immediate PRP group at 4 weeks, supporting the hypothesis that early intervention during the degenerative process may be more effective than treatment of advanced disc degeneration. Our findings are consistent with these observations, particularly the relative preservation of NP quantitative MRI parameters in PRP-treated discs.

Regarding MRI-based assessment, quantitative relaxation mapping offers a noninvasive method to monitor disc composition over time. Prior studies using T2 relaxation time after PRP therapy have reported mixed results, possibly because of differences in species, degeneration models, PRP preparation, follow-up duration, and ROI methodology. The present study incorporated multiparametric quantitative MRI, including UTE-derived metrics and both T1 and T2 mapping, allowing longitudinal assessment of AF and NP changes. In our model, T1 and T2 mapping were more sensitive than UTE SNR for identifying differences between treated and untreated discs, suggesting that relaxation mapping may be more appropriate for monitoring early biochemical modulation after biologic therapy.

Human in vivo data remain limited. A prospective, double-blind, randomized controlled trial reported improvements in pain, physical function, and patient satisfaction after intradiscal PRP compared with controls over short-term follow-up, without major safety concerns [6]. Nevertheless, clinical evidence remains insufficient to conclude that PRP induces disc regeneration. The translational value of our study lies mainly in demonstrating the feasibility of quantitative MRI biomarkers for monitoring intradiscal biologic therapy in a controlled preclinical setting.

The imaging findings are consistent with the known anatomy and pathophysiology of the disc–vertebral complex (DVC) [7,8]. The intervertebral disc comprises a collagen-rich annulus fibrosus and a hydrated, proteoglycan-rich nucleus pulposus that resists axial compression. Disc degeneration is characterized by progressive proteoglycan and aggrecan loss, reduced hydration, disc-height loss, and annular disruption. Decreased NP T1 and T2 values in untreated discs are consistent with dehydration and matrix degradation, whereas increased AF T2 values may reflect annular disruption and structural disorganization. The relative preservation of these parameters in PRP-treated discs suggests attenuation of these processes rather than complete restoration of normal disc architecture.

The lower discriminatory performance of UTE-derived SNR compared with T1 and T2 mapping may be explained by the type of tissue changes induced in this model. UTE imaging is particularly valuable for detecting very short T2 components and collagen-rich tissues, including the annulus fibrosus, endplates, and subchondral bone. In the present model, however, the dominant early changes were likely related to hydration loss, proteoglycan depletion, and matrix disorganization within the NP and AF. These compositional changes directly influence relaxation behavior and are therefore better captured by T1 and T2 mapping, as T1 and T2 mapping have been known to reflect tissue microstructure and are highly sensitive to water content.

From a biophysical perspective, the decrease in NP T2 observed in untreated degenerated discs is consistent with reduced water content and proteoglycan depletion, whereas preservation of NP T2 after PRP injection suggests attenuation of dehydration and matrix breakdown. T1 changes may reflect complementary modifications in tissue water mobility, macromolecular environment, and extracellular matrix composition. The relative preservation of T1 and T2 values in PRP-treated discs should therefore be interpreted as a potential protective or modulatory effect on early degenerative changes rather than as proof of true structural regeneration.

Translation to human disc disease requires caution. Rat caudal discs differ substantially from human lumbar discs in size, loading environment, biomechanics, cellular composition, diffusion distances, and tempo of degeneration. In addition, the mechanical injury combined with collagenase injection used here is an aggressive and rapidly progressive model, whereas human degenerative disc disease usually develops over years through multifactorial mechanical, inflammatory, metabolic, and age-related mechanisms. Consequently, the present findings should be regarded as preclinical proof-of-concept data. Further validation will require larger animal cohorts, sham-injection and vehicle-control groups, longer follow-up, standardized PRP characterization, dose–response experiments, and ultimately controlled clinical trials using harmonized imaging and clinical outcomes.

Finally, advanced computational approaches may further enhance the analysis of quantitative MRI and histologic datasets in future work. Deep learning and transfer-learning strategies have already been applied to musculoskeletal MRI tasks, such as automated detection of knee synovial fluid, and data-driven fusion frameworks have also been proposed for biomedical image-based cell identification [23,24]. Although such methods were beyond the scope of this exploratory study, they could be used in future larger datasets to automate disc segmentation, reduce observer variability, integrate multiparametric MRI and histologic information, and identify imaging signatures predictive of biologic treatment response.

Several limitations should be acknowledged. First, the number of animals was small (n = 8), which limits statistical power and precludes definitive conclusions. The study was designed as a preliminary exploratory experiment in accordance with animal-research reduction principles, and longitudinal repeated MRI measurements were used to increase within-subject information. Nevertheless, larger studies, ideally including at least 20 animals or an adequately powered sample-size calculation, are required to confirm these findings. Second, the study did not include a true sham-injection control group or an active comparator treatment. Therefore, the specific biological effect of PRP cannot be fully separated from the potential effects of injection, disc puncture, or natural evolution after injury. Third, PRP characterization was incomplete: platelet concentration was measured, but growth-factor content, activation status, leukocyte subpopulations, and inter-sample biological variability were not assessed. Fourth, the mechanical and collagenase-induced degeneration model is aggressive and reproducible but does not fully reproduce the slower multifactorial pathophysiology of human DDD. Fifth, the follow-up duration remains relatively short and does not allow conclusions regarding long-term regeneration, structural restoration, or durability of treatment effect. Finally, although image analysis was performed by experienced readers using standardized ROI placement, formal interobserver variability and reproducibility analyses were not performed. In addition, no formal power analysis was performed, and the small sample size increases the risk of type II error and limits assessment of reproducibility. The absence of growth factor and cytokine profiling limits interpretation of the precise biologic mechanisms underlying the observed imaging changes. These limitations have been explicitly acknowledged to avoid overinterpretation of therapeutic efficacy.

In conclusion, intradiscal PRP injection appears feasible in this rat DDD model and was associated with attenuation of early degenerative changes assessed by quantitative T1 and T2 mapping and histology. These preliminary findings support the value of quantitative MRI as a noninvasive biomarker for monitoring biologic therapies in preclinical disc degeneration models. However, the results should be considered exploratory. Further adequately powered preclinical studies with standardized PRP characterization, sham and comparator groups, longer follow-up, and reproducibility assessment are required before clinical translation can be supported.

## Data Availability

Data supporting the reported results can be provided by the authors upon request.
